# A High-Throughput Metabolic Microarray Assay Reveals Antibacterial Effects of Black and Red Raspberries and Blackberries against *Helicobacter pylori* Infection

**DOI:** 10.3390/antibiotics10070845

**Published:** 2021-07-12

**Authors:** Candace Goodman, Katrina N. Lyon, Aitana Scotto, Cyra Smith, Thomas A. Sebrell, Andrew B. Gentry, Ganesh Bala, Gary D. Stoner, Diane Bimczok

**Affiliations:** 1 Department of Chemistry and Biochemistry, Montana State University, Bozeman, MT 59717, USA; candace.goodman@montana.edu (C.G.); b.narayanaganesh@gmail.com (G.B.); 2 Department of Microbiology and Cell Biology, Montana State University, Bozeman, MT 59717, USA; KatrinaLyon@montana.edu (K.N.L.); aitanam96@gmail.com (A.S.); cyrasmith23@gmail.com (C.S.); andysebrell@montana.edu (T.A.S.); Gary.Stoner@osumc.edu (G.D.S.); 3 Bozeman Health GI Clinic, Bozeman Health Deaconess Hospital, Bozeman, MT 59715, USA; agentry@bozemanhealth.org

**Keywords:** antibiotic, high-throughput assay, *H. pylori*, anthocyanin, berry, organoid

## Abstract

*Helicobacter pylori* infection is commonly treated with a combination of antibiotics and proton pump inhibitors. However, since *H. pylori* is becoming increasingly resistant to standard antibiotic regimens, novel treatment strategies are needed. Previous studies have demonstrated that black and red berries may have antibacterial properties. Therefore, we analyzed the antibacterial effects of black and red raspberries and blackberries on *H. pylori*. Freeze-dried powders and organic extracts from black and red raspberries and blackberries were prepared, and high-performance liquid chromatography was used to measure the concentrations of anthocyanins, which are considered the major active ingredients. To monitor antibiotic effects of the berry preparations on *H. pylori*, a high-throughput metabolic growth assay based on the Biolog system was developed and validated with the antibiotic metronidazole. Biocompatibility was analyzed using human gastric organoids. All berry preparations tested had significant bactericidal effects in vitro, with MIC_90_ values ranging from 0.49 to 4.17%. Antimicrobial activity was higher for extracts than powders and appeared to be independent of the anthocyanin concentration. Importantly, human gastric epithelial cell viability was not negatively impacted by black raspberry extract applied at the concentration required for complete bacterial growth inhibition. Our data suggest that black and red raspberry and blackberry extracts may have potential applications in the treatment and prevention of *H. pylori* infection but differ widely in their MICs. Moreover, we demonstrate that the Biolog metabolic assay is suitable for high-throughput antimicrobial susceptibility screening of *H. pylori*.

## 1. Introduction

*Helicobacter pylori* is the major cause of human gastric disease worldwide [1,2]. *H. pylori* is an acid-resistant, Gram-negative bacterium that persistently infects the gastric mucosa of approximately half the world’s population, leading to chronic active gastritis [1]. A proportion of infected individuals also develop peptic ulcer disease, autoimmune gastritis or gastric adenocarcinoma, the second leading cause of cancer-related mortality [3]. In spite of decades of active research, no effective vaccine to prevent *H. pylori*-associated illnesses has been developed [4]. Once diagnosed, *H. pylori* infection is generally treated with a combination of antibiotics and proton pump inhibitors. However, increased resistance to two of the standard antibiotics included in *H. pylori* treatment regimens, clarithromycin and metronidazole, has been reported in multiple studies, with resistance rates ranging from 22 to 80% [5,6]. Recently, clarithromycin-resistant *H. pylori* was included in the WHO’s high-priority pathogens list for research and development of new antibiotics [7]. Moreover, poor patient compliance with complex medication regimens contributes to decreased treatment success [8,9]. Therefore, eradication rates of *H. pylori* have dropped below 75% in several countries [10,11]. The high failure rate of traditional *H. pylori* therapies points to an urgent need for novel alternative treatments or preventative strategies to combat *H. pylori* infection [12]. 

A significant body of research in recent years has shown that natural dietary components, especially plants, contain many bioactive compounds—neutraceuticals—with antibacterial effects [13,14,15]. Multiple different berries and their products show significant antimicrobial activity in vitro and in vivo, and some promising studies suggesting effectiveness against *H. pylori* have been published. Thus, data by Chatterjee et al. [16] showed significant inhibition of *H. pylori* growth in the presence of extracts from raspberry, strawberry, cranberry, elderberry, blueberry and bilberry. In another recent study, extracts from unripe Korean raspberries and elm tree bark used in combination significantly suppressed *H. pylori* growth both in vitro and in a mouse model [17]. Amongst the multiple bioactive natural compounds, anthocyanins in colored berries of the genus *Rubus* have attracted special attention. Anthocyanins are glycosylated, water-soluble phenolic compounds that are responsible for the red, purple and blue coloring of multiple berry species [14]. Anthocyanins are strong antioxidants that have been used successfully in cancer chemoprevention models [18] and that have been implicated in the antibacterial activities of berry preparations [19,20]. In an in vitro model of *H. pylori* infection, the anthocyanin cyanidin 3-*O*-glucoside significantly decreased *H. pylori*-induced cell death [21]. Since anthocyanin-containing berry products also have proven anti-inflammatory effects and are stable under acidic conditions [22,23], their potential application in gastric *H. pylori* infection is particularly attractive. 

In our study, we developed a high-throughput metabolic assay to screen different black raspberry, red raspberry and blackberry preparations for their ability to prevent *H. pylori* growth in vitro. In addition, a gastric organoid model was used to evaluate the biocompatibility of black raspberry extract. Our results demonstrate that all berry powders and extracts tested caused a significant reduction in *H. pylori* growth in two different strains at concentrations between 0.5 and 3%. An optimum preparation of black raspberry extract used at 0.5% led to complete inhibition of *H. pylori* growth but did not affect the viability of primary gastric epithelial cells. These results suggest that preparations from black and red raspberries and blackberries have potential as novel antimicrobial agents to combat *H. pylori* infection. 

## 2. Results

### 2.1. Analysis of Powders and Extracts of Black and Red Raspberries and Blackberries for Anthocyanin Content and Composition

**Figure 1:**
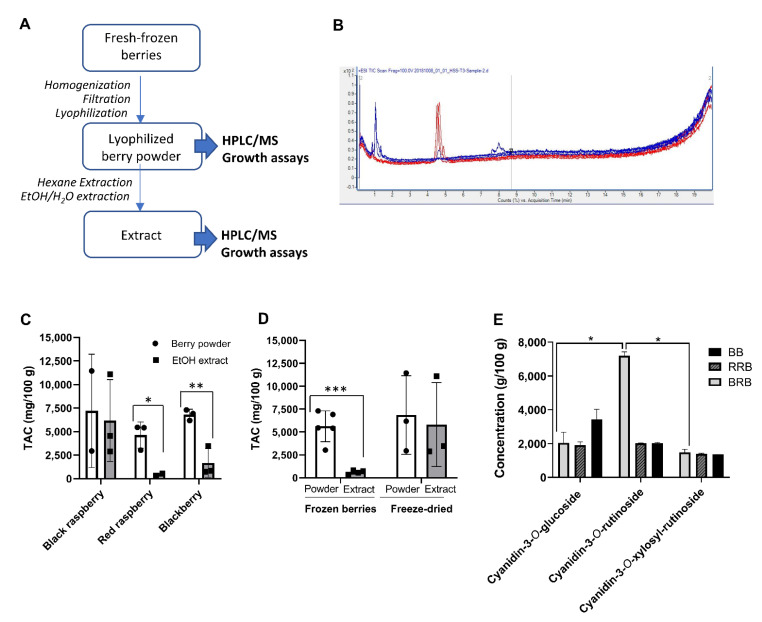
Preparation and anthocyanin content analysis of black raspberries, red raspberries and blackberries. (**A**) Workflow for berry preparation and analysis. (**B**) Representative LC-MS spectrum of a berry preparation. Major peaks represent cyanidin-3-*O*-glucoside, cyanidin-3-*O*-rutinoside, and a combination of cyanidin-3-*O*-xylosylrutinoside and cyanidin-3-*O*-sambubioside. (**C**) Total anthocyanin content (TAC) in suspended powders and ethanol extracts of black raspberries (BRB), red raspberries (RRB) and blackberries (BB) determined by LC-MS. Individual data points, mean and SD are shown. (**D**) TAC in powders and extracts of BRB, RRB and BB purchased as fresh-frozen berries or as freeze-dried berry powder. Pooled data from all berries; individual data points, mean ± SD are shown. (**E**) Concentrations of major anthocyanins in suspended powders of BRB, RRB and BB. Statistically significant differences as determined by (**C**,**D**) Student’s t test (**E**) or two-way ANOVA are shown as * *p* < 0.05, ** *p* < 0.01 and *** *p* < 0.001.

In order to study the potential antibacterial effects of black raspberry (BRB), red raspberry (RRB) and blackberry (BB) compounds on *H. pylori*, freeze-dried berry powders were purchased from different suppliers or were prepared in our laboratory from fresh-frozen berries. Organic extracts of all berry powders were then prepared using hexane/ethanol extraction. The workflow for sample preparation is shown in Figure 1A, and the different starting materials used are listed in Table 1. 

To determine the concentration of anthocyanins, all samples were analyzed by LC–MS for the presence of keracyanin (cyanidin-3-*O*-rutinoside), kuromanin (cyanidin-3-*O*-glucoside) and cyanidin-3-*O*-xylosyrutinoside (Table 1 and Figure 1A,B). Because of overlapping peaks, the cyanidin-3-*O*-xylosyrutinoside may include cyanidin-3-*O*-sambubioside, another phenolic berry compound that has a similar composition and MW as the xylosylrutinoside, and that is known to be present in BRB at a low concentration [24]. 

Total anthocyanin content (TAC) was calculated by adding up the concentrations of all detected anthocyanins (Figure 1C and Table 1). Overall, large variations in anthocyanin concentrations were observed for berries from different sources and with different processing techniques. Interestingly, lyophilized but otherwise untreated berry powder from RRB and BB contained significantly higher amounts of anthocyanins than the water/ethanol extracts prepared in our laboratory (Figure 1C). This was likely due to an inefficient recovery of anthocyanins in extracts prepared from fresh-frozen berries that were lyophilized in-house (Figure 1D, *p* < 0.001, Student’s *t* test), because anthocyanin recovery was higher if extracts were prepared from commercial berry powders (Figure 1D). Individual data for cyanidin-3-*O*-glucoside, cyanidin-3-*O*-rutinoside, and cyanidin-3-*O*-xylosylrutinoside are presented in Figure 1E and Table 2. Notably, BRB, RRB and BB powders contained similar levels of cyanidin-3-*O*-sambubioside and cyanidin-3-*O*-glucoside, but cyanidin-3-*O*-rutinoside levels were significantly higher in BRBs than in RRBs and BBs, as previously described (Figure 1E, *p* < 0.05, mixed model ANOVA). 

### 2.2. Development and Validation of a High-Throughput Assay to Measure H. pylori Growth

**Figure 2:**
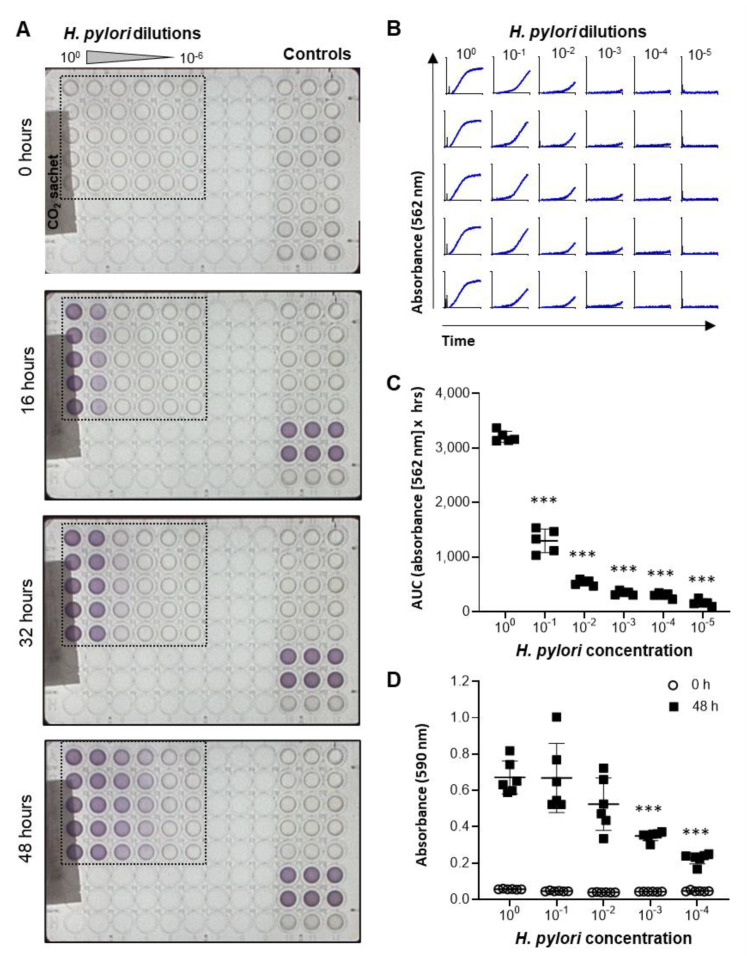
Development of a high-throughput metabolic assay to measure *H. pylori* growth. (**A**) Images of 96-well plates containing various concentrations of *H. pylori* (100 = stock solution used at OD600 = 0.5) obtained by the OmniLog^®^ incubator/reader at different time points after plating the bacteria. (**B**) Growth curves based on absorbance at 562 nm for the wells outlined in panel A. (**C**) Area under the curve was determined using GraphPad Prism and shows significant differences in *H. pylori* metabolism between cultures with different initial concentrations of bacteria. (**D**) Endpoint absorbance (48 h) of an *H. pylori* culture analyzed in a standard 96-well plate reader at 590 nm. Data are representative of *n* = 4 similar experiments with 5–6 technical replicates each. Individual datapoints, mean ± SD are shown. *** *p*< 0.01 compared to undiluted bacteria, one-way ANOVA with Dunnett’s multiple comparisons.

A metabolic bacterial growth assay based on the Biolog system was developed to test a large number of different berry products at different concentrations. This system enables kinetic analysis of microbial growth in a 96-well format based on detection of a redox-sensitive dye by the OmniLog^®^ incubator-reader [25]. Since optimal *H. pylori* growth requires microaerophilic conditions, the 96-well plates were sealed into a plastic sleeve with a CO_2_ Gen Compact sachet to reduce oxygen levels. As shown in Figure 2A and the Supplemental Video S1, addition of *H. pylori* bacteria to the plates at different dilutions resulted in a dose-dependent color change over 48 h. Growth curves had a typical appearance, with an exponential growth phase followed by a plateau phase (Figure 2B). Area under the curve measurements showed significant differences in the growth of *H. pylori* plated at different concentrations, which was confirmed by endpoint measurements at 590 nm in a standard ELISA reader (Figure 2C,D). These results show that *H. pylori* growth can be effectively analyzed in liquid cultures using a high-throughput metabolic growth assay.

### 2.3. Analysis of the Antibacterial Effects of Black and Red Raspberry and Blackberry Powders and Extracts on H. pylori

**Figure 3:**
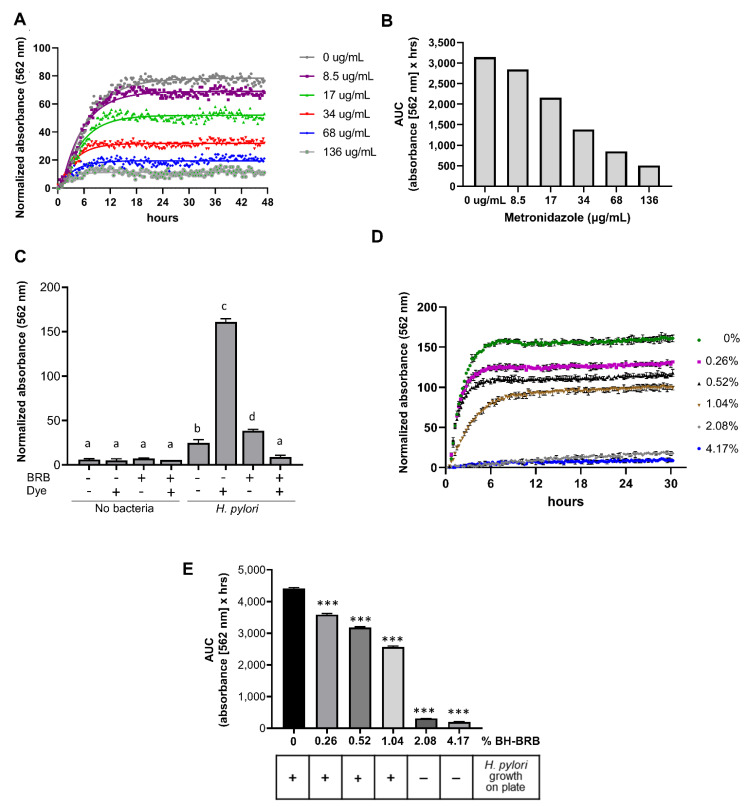
Concentration-dependent growth suppression of *H. pylori* by metronidazole and black raspberries measured using the OmniLog^®^ assay. (**A**) Absorbance of *H. pylori* strain 60190 measured over time in the presence of different concentrations of metronidazole, one representative experiment of three experiments is shown. (**B**) Area under the curve (AUC) values for the experiment shown in (**A**). (**C**) Endpoint absorbance values measured by the OmniLog^®^ after 48 h of culture with and without *H. pylori* strain PMSS1, 8% BH-BRB powder, and/or tetrazolium dye. One representative experiment of three independent experiments with triplicate wells is shown. One-way ANOVA; different letters indicate statistically significant differences at *p* ≤ 0.001. (**D**) Absorbance of *H. pylori* cultures measured over time in the presence of different concentrations of BH-BRB powder. Mean ± SD of triplicate wells, representative of one out of three experiments. (**E**) AUC values for the experiment shown in (**D**). One-way ANOVA; *** indicates statistically significant differences from the untreated control at *p* ≤ 0.001. Bottom panel: matched *H. pylori* growth data on plates, representative of four independent experiments.

The metabolic growth assay was utilized to determine whether anthocyanin-rich berry extracts would inhibit *H. pylori* growth. Metronidazole, a standard antibiotic commonly used in *H. pylori* treatment regimens [26], was utilized to confirm that the OmniLog^®^ assay successfully detected antimicrobial growth inhibition of *H. pylori*. Metronidazole inhibited *H. pylori* growth in a concentration-dependent manner, with complete growth inhibition achieved at 136 µg/mL (Figure 3A,B). Next, we confirmed that the colored berry extracts did not interfere with dye detection in the OmniLog^®^ assay. As shown in Figure 3C, BRB powder (8%) caused no significant signal over baseline after 48 h, whereas in the presence of both *H. pylori* PMSS1 and the metabolic dye, significant absorbance was measured (*p* ≤ 0.001). Absorbance was significantly decreased in the presence of BRB powder. Growth curves over 30 h revealed a concentration-dependent inhibition of *H. pylori* growth at BRB powder concentrations between 0.26% and 4.17% (Figure 3D). Area under the curve (AUC) calculations for the *H. pylori* growth curves similarly showed a significant, concentration-dependent decrease in bacterial growth beginning at 0.26% of berry powder (Figure 3E). To validate the metabolic growth data, 48 h liquid cultures from the growth experiments were re-plated on Brucella agar plates and analyzed for formation of *H. pylori* colonies. Consistent with the results from the metabolic assay, complete growth inhibition was seen at 2.08% and 4.17% of BRB powder, demonstrating strong antibacterial activity of the blackberries on *H. pylori*, whereas colony formation was observed in the absence of BRBs or with lower BRB concentrations (Figure 3E). These results demonstrate the ability of BRBs to inhibit *H. pylori* growth in vitro and the effectiveness of the OmniLog^®^ assay for evaluating *H. pylori* growth and growth suppression by berry compounds. 

**Figure 4:**
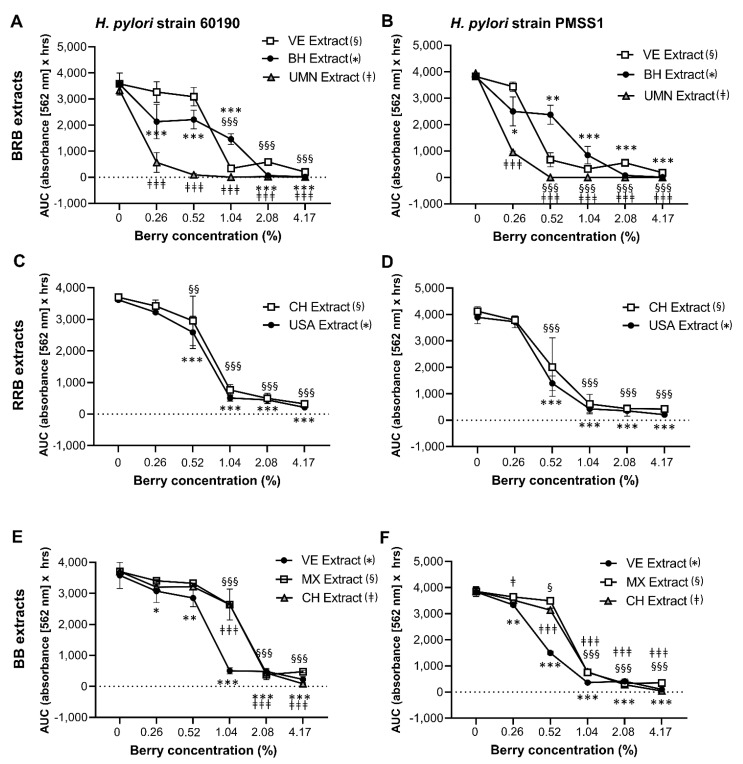
Growth inhibition of *H. pylori* by extracts from BRB, RRB and BB. Growth of *H. pylori* in the presence of different concentrations of the BRB, RRB and BB extracts described in Table 1 and Table 2. Area under the curve (AUC) for metabolic culture activity was measured over 30 h for strain 60190 with (**A**) BRB extracts, (**C**) RRB extracts and (**E**) BB extracts and for strain PMSS1 with (**B**) BRB extracts, (**D**) RRB extracts and (**F**) BB extracts using the OmniLog^®^ assay. Pooled data from *n* = 2–4 independent experiment with 3 technical replicates are shown. Data were analyzed by 2-factorial ANOVA. Tukey’s multiple comparisons test was used to identify differences between a specific concentration of a berry extract and the matching untreated control. Significant differences at *p* ≤ 0.05/*p* ≤ 0.01/*p* ≤ 0.001, respectively, are indicated by */**/***, §/§§/§§§ and ‡/‡‡/‡‡‡ as defined in the panel labels on the right.

**Figure 5:**
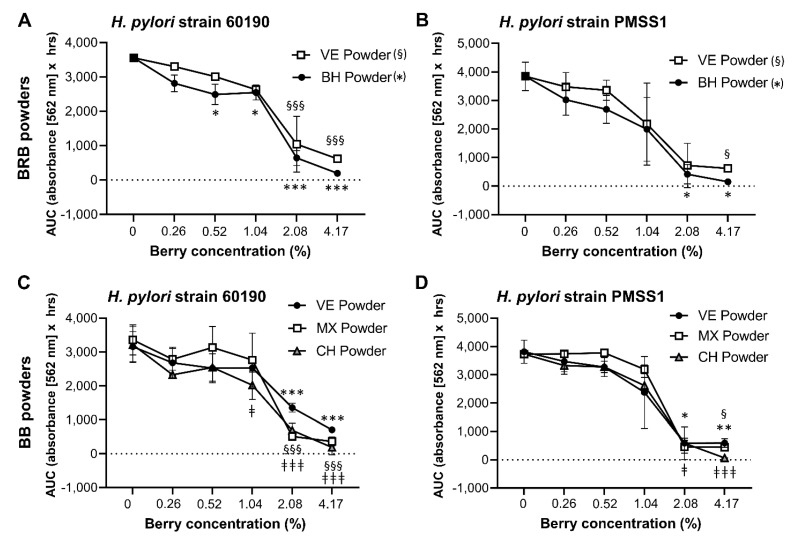
Growth inhibition of *H. pylori* by lyophilized BRB and BB powder. (**A**) Growth of *H. pylori* strains 60190 and PMSS1 in the presence of different concentrations of the BRB and BB powders described in Table 1 and Table 2. Area under the curve (AUC) for metabolic culture activity was measured over 30 h for strain 60190 with (**A**) BRB extracts and (**C**) BB extracts and for strain PMSS1 with (**B**) BRB extracts and (**D**) BB extracts using the OmniLog^®^ assay. Pooled data from *n* = 2–4 independent experiment with 3 technical replicates are shown. Data were analyzed by 2-factorial ANOVA. Tukey’s multiple comparisons test was used to identify differences between a specific concentration of a berry extract and the matching untreated control. Significant differences at *p* ≤ 0.05/*p* ≤ 0.01/*p* ≤ 0.001, respectively, are indicated by */**/***, §/§§/§§§ and ‡/‡‡/‡‡‡ as defined in the panel labels on the right.

As shown in Figure 1 and Table 1 and Table 2, the composition of berry preparations was highly variable, depending on berry species, source and processing method. Therefore, the different BRB, RRB and BB whole berry powders and the extracts described above were dissolved/suspended in culture media and then compared for their ability to inhibit the growth of two well-characterized *H. pylori* strains, 60190 and PMSS1, in the OmniLog^®^ microarray assay. All berry preparations tested had significant antibacterial activity (Figure 4 and Figure 5), with complete inhibition of *H. pylori* growth generally achieved at a concentration of about 4%. However, the different berry preparations showed a great variability in their ability to suppress *H. pylori* growth, with significant effects of the specific preparation identified for extracts from BRB and BB for both *H. pylori* strains (*p* ≤ 0.001) for the RRB extract and the BRB powders for strain 60190 (*p* ≤ 0.05) identified by two-way ANOVA The UMN BRB extract had the strongest antibacterial activity of all the preparations tested, with an MIC_90_ of <0.5%. Conversely, powdered berries generally were less effective than extracts (Figure 5). Both *H. pylori* strains had similar responses to the different berry extracts. 

**Figure 6:**
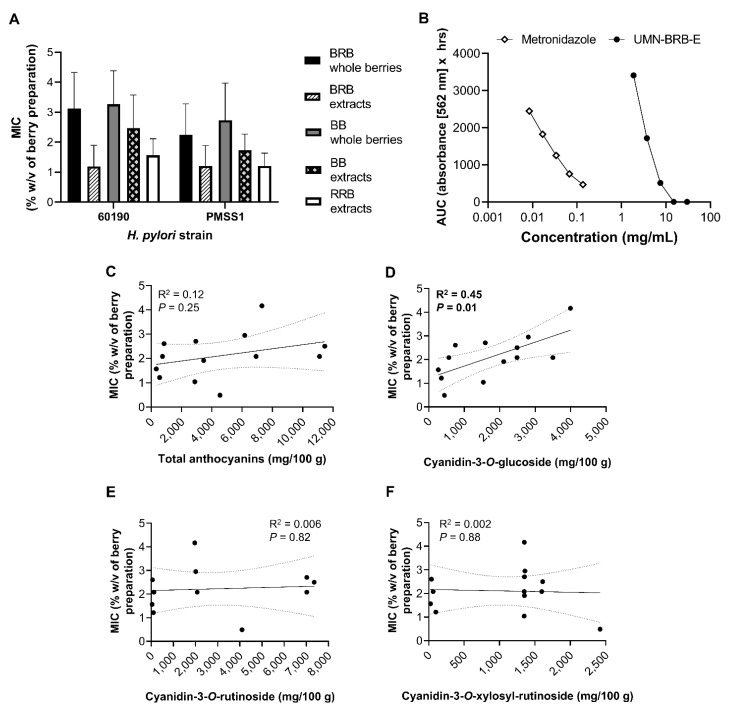
Minimum inhibitory concentrations (MICs) of berry preparations for antibacterial activities against *H. pylori*. (**A**) MIC means ± SD for different berry types and preparations against *H. pylori* strains 60190 and PMSS1. (**B**) Side-by-side comparison of inhibitory effects of metronidazole and *H. pylori* strain 60190; data representative of *n* = 2 independent experiments. (**C**–**F**) Correlation between mean MIC observed with both *H. pylori* strains and concentrations of (**C**) total anthocyanins, (**D**) cyanidin-3-*O*-glucoside, (**E**) cyanidin-3-*O*-rutinoside and (**F**) cyanidin-3-*O*-xylosylrutinoside for all berry preparations analyzed. R^2^ = Pearson’s correlation coefficient.

To better understand the large variability in the antibacterial effects of the different berry preparations, the minimum inhibitory concentrations (MICs) required to suppress *H. pylori* growth were analyzed by multifactorial analysis of variance (ANOVA) of (Figure 6A). Overall, berry extracts exhibited a stronger antibacterial response, as evidenced by significantly lower MICs (*p* ≤ 0.001). For BRB and BB, the type of berry preparation used (powdered lyophilized powder vs. extract) was responsible for 23% of the variation in MIC. The type of berry (BRB, RRB or BB) and the strain of bacteria also significantly impacted MIC, with BRB associated with lower MICs than BB (*p* = 0.019), and strain PMSS1 exhibiting a slightly higher sensitivity to the antibacterial effects of berries than 60190 (*p* = 0.039). Notably, concentrations of berry products required to achieve antibacterial effects were almost two orders of magnitude higher than those observed for metronidazole (Figure 6B). Surprisingly, no significant relationship between the MIC and the total anthocyanin contents or the concentration of cyanidin-3-*O*-rutinoside or cyanidin-3-*O*-xylosylrutinoside was detected based on Pearson’s correlation coefficient (Figure 6C,E,F). In contrast, there was a significant positive correlation between cyanidin-3-*O*-glucoside concentrations and the MICs (Figure 6D, *p* = 0.01, R^2^ = 0.45), indicating that high concentrations of cyanidin-3-*O*-glucoside prevented antibacterial activities. These findings suggest that berry components other than the three anthocyanins analyzed contribute to antibacterial activities of BRB, BB and RRB against *H. pylori*. 

### 2.4. Effect of BRB Extract on Gastric Epithelial Cell Viability in a Human Gastric Organoid Model

**Figure 7:**
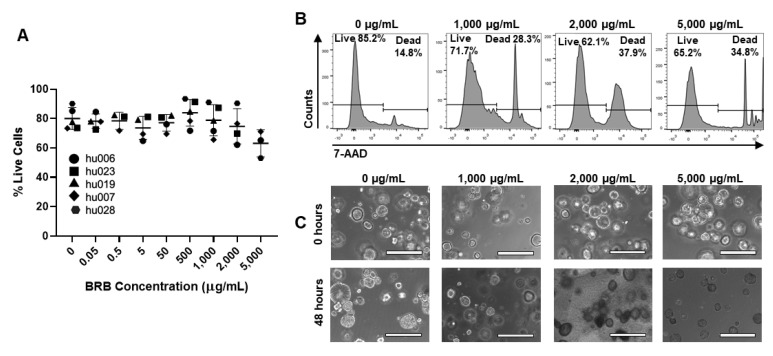
Toxicity analysis of BRB extract in primary human gastric epithelial cell cultures. (**A**) Gastric organoid viability following 48 h of culture in the presence of UMN-BRB extract at different concentrations. Following BRB exposure, cells were harvested by trypsinization and analyzed for 7-AAD dye exclusion by flow cytometry. Pooled data from *n* = 3–5 independent experiments; individual data point, mean ± SD. Differences between treated organoids and untreated controls were analyzed by ANOVA. (**B**) Representative FACS histograms for the data in (**A**) showing 7-AAD staining of gastric organoid cells. (**C**) Representative images of organoid cultures treated with UMN-BRB extract after 0 and 48 h. Bar = 400 µm.

Having shown significant antibacterial effects of multiple different berry preparations against *H. pylori* in vitro, we next sought to confirm that the berries were not toxic to the gastric epithelium, where *H. pylori* bacteria generally reside. Five gastroid lines derived from non-*H. pylori*-infected human gastric biopsies or surgical material were cultured in the presence of UMN-BRB extract, which had the greatest antibacterial activity of all berry preparations tested (Figure 4). As shown in Figure 7, the organoids tolerated the BRB extract over a wide range of concentrations up to 5 mg/mL (0.5%) without any significant impact on organoid viability, as determined by flow cytometric analysis of 7-AAD staining and phase contrast microscopy. 

## 3. Discussion

In this study, significant antimicrobial activity of black and red raspberry and blackberry preparations against *H. pylori* was demonstrated in a high-throughput bacterial growth assay. Interestingly, our analyses showed that both the chemical composition and antimicrobial activity were highly variable depending on berry type, origin and processing method. 

One major advancement of our study was the development of a high-throughput metabolic microarray assay compatible with the growth requirements of the microaerophilic bacterium *H. pylori*, which enabled us to test a large number of different berry preparations at a wide range of concentrations. The efficacy of antimicrobial treatments for *H. pylori* is typically still analyzed using the agar dilution or E strip diffusion methods [27,28,29,30], which are work intensive and not easy to scale up. In a previous study, Lee et al. utilized proprietary Biolog Phenotype Microarray plates to evaluate the ability of *H. pylori* to metabolize different carbon sources [31]. Here, Biolog’s tetrazolium dye and media were used together with metronidazole and various berry dilutions on standard 96- well plates to dynamically analyze *H. pylori* growth inhibition. By sealing liquid *H. pylori* cultures in transparent, gas-impermeable plastic sleeves with small CO_2_ sachets, microaerophilic growth conditions were maintained. Importantly, microarray culture results matched growth profiles on standard agar plates, as demonstrated by re-culturing *H. pylori* samples from the microarray plates. The presence of colored berry preparations did not interfere with dye detection, suggesting that our analysis method is suitable for use with other colored natural products as well as a wide range of other chemical compounds. 

The metabolic growth assay revealed that black and red raspberries and blackberries have the capacity to block *H. pylori* growth in vitro. Antimicrobial properties of *Rubus* berries have been described in multiple previous studies [15,32,33,34,35]. Our analysis of powders and extracts from BRB, RRB and BB from various suppliers and geographical regions revealed that all berry preparations had significant antimicrobial activity against *H. pylori* in vitro, regardless of the *H. pylori* strain used. However, our analyses showed that both the chemical composition and antimicrobial activity (MIC_90_) were highly variable depending on berry type, origin and processing method, corroborating with data from previous studies. Concentrations of active ingredients in *Rubus* berries are known to vary based on geographical location, environmental conditions and berry type [34,36]. In addition, post-harvest processing and storage may affect active ingredients [37,38]. Moreover, different bacterial species vary in their susceptibility to the antibacterial effects of berries [39]. One additional consideration that was not tested here is that consumption of berries or berry products can impact other bacteria in the gastrointestinal tract [40], which in turn might impact *H. pylori* growth [41]. Our data indicate that organic extracts were more potent antimicrobials than powdered berries. Overall, these previous studies and our data indicate that each berry product needs to be carefully tested for specific biological activity and applications prior to use as a neutraceutical. The top-performing preparation in our study was a black raspberry extract from the University of Minnesota (UMB-BRB-E), which achieved complete *H. pylori* growth inhibition at (>90%) at 0.5% (5 mg/mL). This inhibitory concentration is similar to or lower than those of *Rubus* extracts described in other studies [16,32,42], but several log folds higher than standard antibiotics such as amoxicillin and clarithromycin [43] or the metronidazole used in our study, which completely blocked *H. pylori* growth at 136 µg/mL.

Since anthocyanins are considered the major active ingredients of black and red berries, we hypothesized that anthocyanins would also be responsible for the antibacterial activities observed in our experiments. It was expected that correlation analysis would show a significant inverse relationship between the anthocyanin concentration and the MIC of the berry preparations. Surprisingly, no significant correlation between the concentration of any anthocyanin analyzed in our preparations and increased antibacterial activity was detected, indicating that antimicrobial activity was largely independent of anthocyanins. Anthocyanins exhibit antimicrobial activity against Gram-negative bacteria by causing damage to the cell walls, membranes, and intercellular matrix [44]. Importantly, anthocyanins have been linked to the antimicrobial effects of berry preparations in previous studies [45,46,47] and are responsible for the major chemopreventive effects of blackberries and black and red raspberries [18,24,48,49,50]. However, our correlation analysis suggests that the antibacterial effects against *H. pylori* were independent of anthocyanins and thus must be caused by other active compounds. Indeed, ellagic acid, another polyphenol present in red and black berries, is known to exert antibacterial activity against *H. pylori* as well as other bacteria [17,51,52]. In addition, Lengsfeld et al. demonstrated that berry-derived polysaccharides can combat *H. pylori* infection in vivo by preventing bacterial binding to the gastric mucosa [53]. Additional studies have shown antibacterial effects for berry-derived sanguiin H-6 [35] and rubusoside [54]. Further experiments are needed to identify the raspberry and blackberry compounds that mediate antimicrobial activity against *H. pylori*. 

It remains to be tested whether BRB extract can be used to successfully treat *H. pylori* infection in vivo. Berry compounds have been investigated in many studies, and their pharmacokinetics and pharmacodynamics have previously been characterized [51,55]. In our study, human gastric organoids were used as model of primary human gastric epithelial cells to analyze compatibility of BRB extract with gastric epithelial cells, since they closely represent the architecture and cellular complexity of the human gastric mucosa [56,57]. Organoids are three-dimensional long-term cultures of primary cells maintained in a gelatinous extracellular matrix in the presence of specific growth factors. Importantly, exposure of the organoids to BRB did not negatively impact cell viability at the concentrations tested. Moreover, previous animal studies aimed at characterizing the chemopreventive properties of berries have demonstrated that berry products including powdered BRBs are well tolerated at dietary concentrations of up to 10% [58] and that supplementation of the diet with 5% BRB powder, equivalent to 45 g/day in humans [59], prevented esophageal, oral and colon cancer in rats and colonic polyps in mice [48,60,61]. In a phase I clinical trial, administration of 60 g of BRB powder per day had beneficial effects in colorectal cancer patients with no significant side effects except transient diarrhea or constipation [62]. Other studies have demonstrated that berry extracts have anti-*Helicobacter* activity in animal models. Thus, Park et al. [17] recently showed that extracts prepared from dried, unripened Korean raspberry (*Rubus crataegifolius*) decreased *H. pylori* colonization by about 4 log-fold in a murine model of infection with *H. pylori* strain SS1. Notably, the berry preparation used in the study by Park et al. was highly potent, with an in vitro MIC_90_ of 150 µg/mL [17]. 

In summary, we have established a high-throughput metabolic growth assay to analyze antimicrobial effects of berry preparations against *H. pylori*. Both freeze-dried powders and ethanol extracts from BRBs, RRBs and BBs obtained from various sources significantly suppressed growth of multiple *H. pylori* strains in vitro. Toxicity studies with human gastric organoids demonstrated good biocompatibility over a wide range of concentrations, including the MIC_90_ determined in the growth assay. Together, our findings confirm the potential of berry products as antimicrobial agents but highlight the importance of carefully selecting specific preparations with high antibacterial activity.

## 4. Materials and Methods

### 4.1. Berry Powders and Preparation of Extracts

Commercially available black raspberry (*Rubus occidentalis*; BRB), blackberry (*Rubus fruticosus*; BB) and red raspberry (*Rubus idaeus*; RRB) samples were either purchased as freeze-dried powders or fresh-frozen whole berries. One additional BRB extract prepared as described in previous studies in Dr. S. Hecht’ laboratory at the University of Minnesota was included for comparison [50,63,64] Suppliers and countries of origin for the berries are listed in Table 1. Fresh-frozen whole berries were processed into freeze-dried powder using a food processor (Cuisinart^®^ Elemental, Stamfort, CT, USA) and lyophilizer (SP VirTis Genesis Pilot Lyophilizer, Warminster, PA, USA) (Figure 1A). Berry powders were either used directly in experiments after mixing the material with IF-10a media plus dye D, both Biolog, Hayward, CA, USA at a final concentration of 0.26–4.17% (*w*/*v*; powders) or were processed for hexane/ethanol extraction (extracts). Notably, berry powders contain both water soluble and insoluble materials, so that mixing of the powders with aqueous media results in a colloidal suspension. For hexane/ethanol extraction, nonpolar compounds were extracted using 3 × 100 mL hexane per 10 g powder. Samples were filtered between each extraction, and the hexane filtrate discarded. Anthocyanins and all water-soluble compounds were then extracted using an 80:20 ethanol:water mixture (3 × 100 mL per 10 g sample). This extract was dried to a syrup under reduced pressure at 30 °C then lyophilized to yield between 1 and 4 g per 20 g powder. All berry preparations were stored in airtight containers at −20 °C until use. 

### 4.2. Analysis of Anthocyanin Content

Concentrations of major active compounds, i.e., cyanidin-3-*O*-glucoside, cyanidin-3-*O*-rutinoside, cyanidin-3-*O*-xylosyl-rutinoside and cyanidin-3-sambubioside, for both powder and extract samples were measured by HPLC–MS. The lyophilized samples were mixed with 80:20 (water: sample). The samples were filtered and then injected into an LC–MS system (Agilent 6538 UHD-QTOF equipped with Agilent 1290 infinity UPLC). Upon extracting the chromatograms based on the reported *m*/*z*, calculations were performed by integrating the peak to obtain the area. Anthocyanin standards were purchased from Extrasynthese S.A.S. (Lyon, France).

### 4.3. Helicobacter pylori Strains and Culture Conditions

Two well-characterized *cagA^+^*, *vacA s1/m1 H. pylori* strains, originally isolated from human patients, were used in our experiments: the reference strain 60190 (kind gift from Dr. G. Perez-Perez, New York University, ATCC #49503 [65]), and strain PMSS1 (kind gift from Dr. K. Wilson, Vanderbilt University), which is widely used in murine infection experiments [66]. *H. pylori* strain 60190 was shown to be susceptible to metronidazole, amoxicillin, clarithromycin, levofloxacin, rifampicin and tetracycline [67], and strain PMSS1 was confirmed to be susceptible to metronidazole, amoxicillin, clarithromycin, and tetracycline in previous studies [68,69]. For the experiments, bacteria were grown at 37 °C under microaerophilic conditions on Brucella agar plates, 5% sheep blood (Becton Dickinson) for 3 days. Colonies were harvested into warm Brucella broth supplemented with 10% FBS and were then cultured in a shaking incubator for a further 18 h period prior to use in the experiments. 

### 4.4. High-Throughput Helicobacter pylori Growth Assay

High-throughput bacterial growth assays were performed in 96-well plates using an OmniLog (Biolog, Hayward, CA, USA) plate reader-incubator. Bacterial growth was visualized with a proprietary redox-sensitive tetrazolium dye [70] (dye D, Biolog). Serial dilutions of berry suspensions or extracts (0.26–4.17% *w*/*v*) or of metronidazole (8.5–136 µg/mL; Acros Organics, Fair Lawn, NJ, USA) prepared in IF-10a were added to the plates as indicated together with dye D (0.01%) and PM additive (0.05% BSA, 0.01% NaHCO_3_ and 0.045% glucose *w*/*v* final concentrations). Live *H. pylori* was resuspended in IF-10a (Biolog) to a final OD600 = 0.5, which corresponds to 3.4 × 10^8^ bacteria/mL [71], and 20 µL of the bacterial suspension were added to the plates together with berry preparations at appropriate dilutions, for a total volume of 120 µL. For analysis, loaded plates were sealed in a gas-impermeable bag with a CO_2_ Compact sachet (Oxoid, Nepean, ON, Canada), following the manufacturer’s instructions for culturing of microaerophilic bacteria within the OmniLog incubator-reader. Using the bags and sachets was necessary to create microaerophilic conditions, since the OmniLog does not have a controlled CO_2_ atmosphere. Plates then were incubated at 37 °C in the OmniLog incubator for 30–48 h. Absorbance values were recorded at 562 nm every 15 min. In some instances, 10-fold serial dilutions of the *H. pylori* cultures were recovered from the plates and were re-streaked on Brucella agar plates to confirm growth and growth suppression.

### 4.5. Data Analysis for Bacterial Growth Assays

To analyze *H. pylori* growth inhibition by berry compounds, OmniLog data were exported to Excel using the Biolog Data Converter (version 1.0) and PM Analysis Software (Microbe, version 1.20.02, all Biolog, Hayward, CA, USA). Absorption data for each sample and time point were normalized to baseline by subtracting the average of the first four absorption values from each data point. To quantitate bacterial growth over time, peak area under the curve (AUC; absorbance [562 nm] × h) was determined using GraphPad version 8.3.1 (San Diego, CA, USA). The minimum inhibitory concentration 90 (MIC_90_) of a berry preparation was defined as the concentration at which the AUC values for the growth curves were decreased to ≤10% of the maximum. 

### 4.6. Human Gastric Organoid Culture and Viability Assay

Human gastric organoid cultures (gastroids) were established and maintained as previously described [72,73]. Briefly, human gastric tissue samples were obtained with informed consent and IRB approval from patients undergoing endoscopy and biopsy at the Bozeman Health Deaconess Hospital (protocol DB050718-FC). Alternatively, tissue samples from sleeve gastrectomy surgeries were provided by the National Disease Research Interchange (protocol DB062615-EX). None of the donors were positive for active *H. pylori* infection, as determined by rapid urease CLO test (Halyard Health, Alpharetta, GA, USA). Gastric glands were prepared by collagenase digestion and then were plated in Matrigel. Following polymerization, matrigel was overlaid with L-WRN medium which includes Advanced DMEM/F12 (Gibco by Life Technologies, Grand Island, NY, USA) and 50% supernatant from murine L-WRN cells—which secrete Wnt3a, noggin, and R-spondin 3—and supplemented with 10% FBS (Rocky Mountain Bio, Missoula, MT, USA), 1% L-Glutamine, 10µM Y-27632 (Tocris Biosciences, Bristol, UK), 10 µM SB-431542 (Tocris Biosciences, Bristol, UK), and 10 mM HEPES buffer. Black raspberry extract prepared in 90% DMSO and 10% HCl was externally administered to the organoids for a 48 h treatment at 37 °C with 5% CO_2_. Control cultures were treated with dilutions of 90% DMSO/10% HCl alone. To determine cell viability, organoids were harvested by trypsinization, and single-cell suspensions stained with 7-aminoactinomycin D (7-AAD; ThermoFisher Scientific, Waltham, MA, USA) were analyzed on an LSR II flow cytometer (Becton Dickinson). 

### 4.7. Statistical Analysis

Data shown are representative of three or more replicate experiments. For OmniLog assays, 3–6 technical replicates were prepared. All data were analyzed using GraphPad version 8.3.1 (San Diego, CA, USA). Data are shown as the mean ± SD. Student’s *t* test or a one- or two-way ANOVA with Tukey’s or Dunnett’s multiple comparisons test were used to determine statistical significance. Differences were considered significant at *p* ≤ 0.05.

## Figures and Tables

**Table 1:** Total concentrations of anthocyanins in black and red raspberry and blackberry powders and extracts determined by LC–MS.

Sample	Source	Country of Origin	Material Type	HPLC–MS *	
				Berry Powder TAC (mg/100 g)	Extract TAC (mg/100 g) *
VE-BRB	VirginExtracts, Foods Super, Bradford, PA, USA	Unknown	Freeze-dried powder	2945	2885
BH-BRB	BerriHealth, Berri Products LLC, Corbett, OR, USA	USA	Freeze-dried powder	11,455	11,109
UMN-BRB	Dr. S. Hecht, University of Minnesota, Minneapolis, MN, USA	USA	Ethanol extract	N/A	4540
USA-RRB	Great Value. Wal-Mart Stores Inc. Bentonville, AR, USA	USA	Whole frozen berries	5424	554
CH-RRB	Cascadian Farms, Small Planet Foods, Inc., Sedro-Woolley, WA, USA	Chile	Whole frozen berries	3027	336
VE-BB	VirginExtracts, Foods Super, Bradford, PA, USA	Unknown	Freeze-dried powder	6175	3465
MX-BB	Western Family Foods, Inc., Portland, OR, USA	Mexico	Whole frozen berries	6924	748
CH-BB	Cascadian Farms, Small Planet Foods, Inc., Sedro-Woolley, WA, USA	Chile	Whole frozen berries	7312	847

* Absorption assumes all anthocyanins are cyanidin-3-glucoside equivalents (sum of three major cyanidins). BRB: black raspberry; RRB: red raspberry; BB: blackberry; VE: VirginExtracts; BH: BerriHealth; UMN: University of Minnesota; CH: Chile; MX: Mexico; TAC: total anthocyanin content; HPLC–MS: high-performance liquid chromatography/mass spectrometry.

**Table 2:** Anthocyanin composition within powdered berries and berry extracts determined by HPLC–MS.

Sample		HPLC–MS * (mg/100 g)		
		Cyanidin-3-*O*-glucoside	Cyanidin-3-*O*-rutinoside	Cyanidin-3-*O*-xylosylrutinoside
VE-BRB	Powder	1593	7025	1352
	Extract	1537	No data	1348
BH-BRB	Powder	2490	7356	1609
	Extract	2487	7025	1597
UMN-BRB	Powder	No data	No data	No data
	Extract	440	4100	2420
USA-RRB	Powder	1999	1993	1432
	Extract	355	98	101
CH-RRB	Powder	1678	No data	1349
	Extract	267	43	26
VE-BB	Powder	2806	2010	1359
	Extract	2113	No data	1352
MX-BB	Powder	3499	2076	1349
	Extract	564	121	63
CH-BB	Powder	3995	1966	1351
	Extract	748	60	39

* BRB: black raspberry; RRB: red raspberry, BB: blackberry; VE: VirginExtracts; BH: BerriHealth; UMN: University of Minnesota; CH: Chile; MX: Mexico; TAC: total anthocyanin content; HPLC–MS: high-performance liquid chromatography/mass spectrometry.

## Data Availability

Original data will be made available by the authors upon reasonable request.

## Supplementary Materials

The following are available online at https://www.mdpi.com/article/10.3390/antibiotics10070845/s1, Video S1: Time lapse series of plate images of the *H. pylori* culture in Figure 2 obtained on the OmniLog™ incubator-reader shows color development consistent with *H. pylori* metabolism and growth over 48 h.
